# The Role of Yeasts in Fermentation Processes

**DOI:** 10.3390/microorganisms8081142

**Published:** 2020-07-28

**Authors:** Sergi Maicas

**Affiliations:** Departament de Microbiologia i Ecologia, Facultat de Ciències Biològiques, Universitat de València, 46100 Burjassot, País Valencià, Spain; sergi.maicas@valencia.edu; Tel.: +34-963543214

**Keywords:** yeast, non-*Saccharomyces* yeast, wine, beer, beverages

## Abstract

In recent years, vessels have been discovered that contain the remains of wine with an age close to 7000 years. It is unclear whether, in ancient times, humans accidentally stumbled across fermented beverages like wine or beer, or was it a product intended as such. What is a fact is that since then, alcoholic beverages have been part of the diet and culture of many of the civilizations that have preceded us. The typical examples of beer and wine are an example of many other drinks resulting from the action of yeasts. In addition to these two beverages, various companies have developed other types of fermented foods and non-alcoholic beverages prepared in a traditional or commercial manner. The climatic conditions, the availability of raw material and the preferences of each region have conditioned and favored the maintenance of some of these products. In addition to the aforementioned traditional alcoholic beverages produced from fruits, berries, or grains, humans use yeast in the production of chemical precursors, global food processing such as coffee and chocolate, or even wastewater processing. Yeast fermentation is not only useful in food manufacturing. Its uses extend to other products of high interest such as the generation of fuel from vegetable sources.

## 1. Introduction

**Figure 1:**
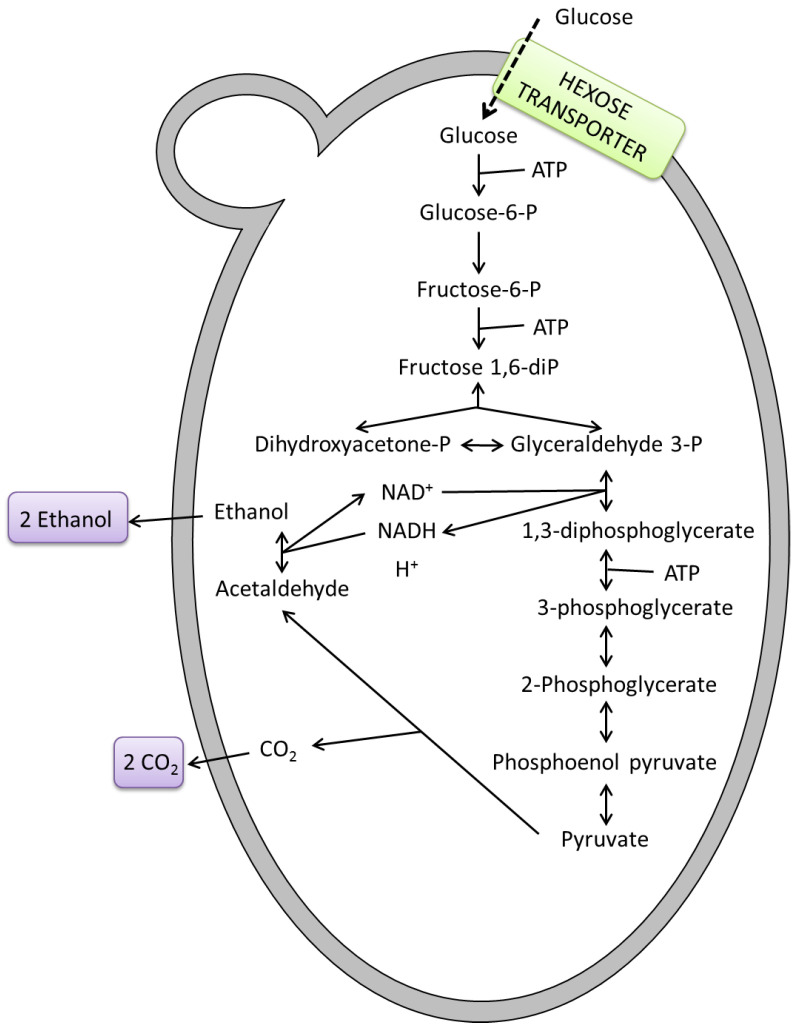
Central metabolism of fermentation in yeasts.

Fermentation is a well-known natural process used by humanity for thousands of years with the fundamental purpose of making alcoholic beverages, as well as bread and by-products. Upon a strictly biochemical point of view, fermentation is a process of central metabolism in which an organism converts a carbohydrate, such as starch or sugar, into an alcohol or an acid. For example, yeast performs fermentation to obtain energy by converting sugar into alcohol. Fermentation processes were spontaneously carried out before the biochemical process was fully understood. In the 1850s and 1860s, the French chemist and microbiologist Louis Pasteur became the first scientist to study fermentation, when he demonstrated that this process was performed by living cells. Fermentation processes to produce wines, beers and ciders are traditionally carried out with *Saccharomyces cerevisiae* strains, the most common and commercially available yeast. They are well known for their fermentative behavior and technological characteristics which allow obtaining products of uniform and standard quality. Many other important industrial products are the result of fermentation, such as yogurt, cheese, bread, coffee. Yeasts also play a key role in wastewater treatment or biofuel production. Upon a biochemical point of view, fermentation is carried out by yeasts (and some bacteria) when pyruvate generated from glucose metabolism is broken into ethanol and carbon dioxide (Figure 1).

The schematic chemical equation for the production of ethanol from glucose is as follows:

$$C_{6}H_{12}O_{6}(\mathrm{glucose})⟶2C_{2}H_{5}\mathrm{OH}(\mathrm{ethanol})+{\mathrm{CO}}_{2}(\mathrm{carbon dioxide})$$

Under absence or oxygen-limited conditions, ethanol is produced from acetaldehyde, and two moles of ATP are generated. This is not a fully satisfactory reaction for cells, as they have to consume high amounts of glucose to deliver enough ATP to the system. As a consequence, ethanol is accumulated and when this occurs the fermentative activity is stopped [1].

### 1.1. Yeasts

Yeasts are eukaryotic microorganisms that live in a wide variety of ecological niches, mainly in water, soil, air and on plant and fruit surfaces. Perhaps the most interesting habitat at this point is the latter, since they directly intervene in the decomposition of ripe fruit and participate in the fermentation process. In this natural environment, yeasts can carry out their metabolism and fermentation activity satisfactorily as they have the necessary nutrients and substrates [2]. On a nutritional level, yeasts are not particularly demanding compared to other microorganisms such as lactic acid bacteria. However, their growth is supported by the existence of basic compounds such as fermentable sugars, amino acids, vitamins, minerals and also oxygen. Upon a morphological point of view, yeasts present a high morphological divergence, with round, ellipsoidal and oval shapes being the most common. In fact, in the identification processes, microscopic evaluation is the first resource followed by other more discriminatory tests such as microbiological and biochemical ones. In a next stage, the classical classification includes other more laborious tests such as those of sugar fermentation and amino acid assimilation [2]. The production and tolerance to ethanol, organic acids and SO${}_{2}$ are also important tools to differentiate among species. The reproduction of yeasts is mainly by budding, which results in a new and genetically identical cell. Budding is the most common type of asexual reproduction, although cell fission is a characteristic of yeasts belonging to the genus *Schizosaccharomyces*. Growing conditions that lead to nutrient starvation, such as lack of amino acids, induce sporulation, which is a mechanism used by yeasts to survive in adverse conditions. As a result of sporulation, yeast cells suffer from genetic variability. In industrial fermentation processes, the asexual reproduction of yeasts is advisable to ensure the preservation of the genotype and to maintain stable fermentation behaviour that does not derive from it for as long as possible. At the metabolic level, yeasts are characterised by their capacity to ferment a high spectrum of sugars, among which glucose, fructose, sucrose, maltose and maltotriose predominate, found both in ripe fruit and in processed cereals. In addition, yeasts tolerate acidic environments with pH values around 3.5 or even less. According to technological convenience, yeasts are divided into two large groups namely *Saccharomyces* and non-*Saccharomyces*. Morphologically, *Saccharomyces* yeasts can be round or ellipsoidal in shape depending on the growth phase and cultivation conditions. *S. cerevisiae* is the most studied species and the most utilized in the fermentation of wines and beers due to its satisfactory fermentative capacity, rapid growth and easy adaptation. They tolerate concentrations of SO${}_{2}$ that normally most non-*Saccharomyces* yeasts do not survive. However, despite these advantages, it is possible to find in the nature representatives of *S. cerevisiae* that do not necessarily have these characteristics.

### 1.2. Non-*Saccharomyces* Yeasts

Non-*Saccharomyces* yeasts are a group of microorganisms used in numerous fermentation processes, since their high metabolic differences allow the synthesis of different final products. Generally, many of these yeasts capable of modifying the sensory quality of wines are considered as contaminants, so eliminating them or keeping them at low levels was a basic objective in the past [3]. In order to eliminate their activity in wine fermentation, it is usual to disinfect the tanks and fermentation containers using sulfite. This perception has been modified year after year, gaining relevance the action of these yeasts in the spontaneous fermentation, since they contribute positively in the final sensory quality of the wine. These yeasts are the majority in the initial phase of spontaneous fermentation to the point where the concentration of ethanol reaches 4 and 5% *v*/*v*. At that point, between alcohol and the exhaustion of dissolved oxygen, their growth is inhibited [4]. When the process is completed, *Saccharomyces* yeasts, the most resistant to ethanol, predominate and complete the fermentation. It has been reported that some non-*Saccharomyces* yeasts are able to survive toward the end of the spontaneous fermentation and exert their metabolic activity, thus contributing positively to the sensory quality of wines. Based on this evidence, in recent years, many researchers have focused their studies in understanding the nature and fermentative activity of the non-*Saccharomyces* yeasts [5]. The findings demonstrated the enormous potential of these yeasts for use in the fermentation of traditional and nontraditional beverages. Despite the fact that most non-*Saccharomyces* yeasts show some technological disadvantages compared to *S. cerevisiae* such as lower fermentative power and production of ethanol, non-*Saccharomyces* yeasts possess characteristics that in *S. cerevisiae* are absent, for instance, production of high levels of aromatic compounds such as esters, higher alcohols and fatty acids [6]. In addition, it has been reported that the fermentative activity of these yeasts is manifested in the presence of small amounts of oxygen which leads to an increase in cell biomass and the decrease in ethanol yield, a strategy that can be used to reduce the ethanol content of wines produced in coculture with *S. cerevisiae* [7]. With the aim of exploiting the positive characteristics of non-*Saccharomyces* yeasts and reducing their negative impact, fermentations with mixed and sequential cultures with *S. cerevisiae* can be performed to produce fermented beverages with different sensory profiles [8]. The most important fact is related to the potential for producing a broad variety of compounds of sensory importance necessary to improve the organoleptic quality of wines and beers. The findings reported so far in literature have led to rethink the role of these yeasts in fermentative processes and to evaluate their use in the development of new products. Among the most studied non-*Saccharomyces* yeasts that reached special importance for researchers include *Candida*, *Kloeckera*, *Hanseniaspora*, *Brettanomyces*, *Pichia*, *Lanchacea* and *Kluyveromyces*, among others.

## 2. Yeast Fermentation Processes

### 2.1. Alcoholic Fermentations

The production of alcoholic beverages from fermentable carbon sources by yeast is the oldest and most economically important of all biotechnologies. Yeast plays a vital role in the production of all alcoholic beverages. Yeast plays a vital role in the production of all alcoholic beverages and the selection of suitable yeast strains is essential not only to maximise alcohol yield, but also to maintain beverage sensory quality [2].

#### 2.1.1. Wine Fermentation

In wine fermentation, strains with specific characteristics are needed, for instance, highly producers of ethanol to reach values of 11–13% *v*/*v*, typically found in this beverage. On the other hand, beers and ciders contain less amounts of ethanol with a balanced and distinctive sensory profile characteristic of each one. In recent years, new consuming trends and requirements for new and innovative products have emerged. This situation led to rethink about the existing fermented beverages and to meet the demands of consumers. Yeasts are largely responsible for the complexity and sensory quality of fermented beverages. Based on this, current studies are mainly focused on the search of new type of yeasts with technological application. Non-*Saccharomyces* yeasts have always been considered contaminants in the manufacture of wine and beer. Therefore, procedures for eliminating them are routinely utilized such as must pasteurization, addition of sulfite and sanitization of equipment and processing halls. In recent years, the negative perception about non-*Saccharomyces* yeasts has been changing due to the fact that several studies have shown that during spontaneous fermentations of wine, these yeasts play an important role in the definition of the sensory quality of the final product. Based on this evidence, the fermentative behavior of some non-*Saccharomyces* yeasts is being studied in deep with the purpose of finding the most adequate conditions and the most suitable strain to be utilized in the production of fermented beverages.

#### 2.1.2. Beer Fermentation

Beer is the most consumed alcoholic beverage worldwide. It is traditionally made from four key ingredients: malted cereals (barley or other), water, hops, and yeast. Each of these ingredients contributes to the final taste and aroma of beer. During fermentation, yeast cells convert cereal-derived sugars into ethanol and CO${}_{2}$. At the same time, hundreds of secondary metabolites that influence the aroma and taste of beer are produced. Variation in these metabolites across different yeast strains is what allows yeast to so uniquely influence beer flavor [9]. Although most breweries use pure yeast cultures for fermentation, spontaneous or mixed fermentation is nowadays used for some specialty beers. These fermentation procedures involve a mix of different yeast species (and bacteria as well) that contribute to the final product sequentially, giving the beer a high degree of complexity. Commonly, breweries have their own stock of selected yeasts for their specific beers. As it is well-known, two types of yeast are used in brewing: *S. cerevisiae* as the top-fermenting yeast to make ales while *S. pastorianus* is a bottom-fermenting yeast used in lager brewing processes [10].

#### 2.1.3. Cider Fermentation

Cider is another alcoholic beverage derived from the apple fruit industry, very popular in different countries in the world, mainly Europe, North America, and Australia [11]. Although traditional ciders are produced from spontaneous fermentation of juice carried out by autochthonous yeasts, selected *S. cerevisiae* strains are also commonly used to carry out alcoholic fermentation. This ensures a consistent quality of the finished products [12]. Some other non-*Saccharomyces* yeast species are involved in spontaneous fermentation of apple juice for cider production. However, these yeasts contribute at a lesser extent than *Saccharomyces* and can be producers of off-flavours [13]. Research articles on this type of product are scarce compared to wine, especially in phenomena associated with microbial activities. The microbiome of wine fermentation and its dynamics, the organoleptic improvement of healthy and pleasant products and the development of starters are now extensively studied. Although the two beverages seem close in terms of microbiome and process (with both alcoholic and malolactic fermentations), the inherent properties of the raw materials and different production and environmental parameters make it worthwhile research on the specificities of apple fermentation. An excellent review of the microbial implications associated with cider production, from ecosystem considerations to associated activities and the influence of process parameters [11].

In addition to these three worldwide-famous fermented beverages, there are many others made from fruit in various countries in Africa, Asia, and Latin America. Although its consumption is local or regional, in some countries drinks made using fruits such as bananas or grapes as raw materials are very popular. The most widespread alcoholic fruit drink in Eastern Africa is banana beer, which in addition to gastronomic interest is especially culturally relevant. Banana beer is a mixed beverage made from bananas and a cereal flour (often sorghum flour) [14]. Dates in North Africa, pineapples and cashew fruits in Latin America and jack fruits in Asia are other of the most relevant products.

### 2.2. Non-Alcoholic Fermentations

Moreover, yeast can act in the fermentation of global non-alcoholic products (bread, chocolate or coffee, beverages such as kefir, sodas, lemonades, and vinegar or even biofuels and other chemicals.

#### 2.2.1. Bread Fermentation

The fermentation of the dough made by the yeasts is the most critical phase in the making of bread. The fermentative yield of yeast cells during this fermentation is crucial and determines the final quality of the bread. Yeasts not only produce CO${}_{2}$ and other metabolites that influence the final appearance of the dough, volume, and texture, and of course, the taste of the bread. The yeast strain, pregrowth conditions, its activity during the dough fermentation process, the fermentation conditions, as well as the dough ingredients are basic to control the process. The fermentation rate is also conditioned by the ingredients of the dough, including the amounts of sugar and salt used in its preparation. Commercial bread producers currently produce various types of dough such as lean, sweet or frozen dough. Depending on the type of dough, and to obtain optimal fermentation rates, it is recommended to use suitable yeast strains with specific phenotypic traits [15].

#### 2.2.2. Coffee Fermentation

Yeasts play an important role in coffee production, in the post-harvest phase. Its performance can be done in two phases. On the one hand, aerobically, in which the berries just collected are deposited in a tank and the yeasts are allowed to act. This process is carried out under control of basic parameters, such as time and temperature. Alternatively, coffee berries are deposited in a container mixed with water and microorganisms are allowed to act anaerobically (in the absence of oxygen). This second process is more homogeneous and easy to control than the aerobic. Sometimes, coffee beans are even fermented in a mixed process, first in an aerobic and finally anaerobic manner [16]. To develop these processes in a satisfactory manner, and to preserve/improve the organoleptic properties of coffee, refine its sweetness, control acidity, give them body or add sensory notes (chocolate, caramel, fruits) mucilage should be removed. The process is naturally carried out by the yeasts present in the mixture, although the process can be improved by the addition of appropriate enzymes (polygalacturonase, pectin lyase, pectin methylesterase) [17].

#### 2.2.3. Chocolate Fermentation

Raw cacao beans have a bitter and astringent taste, because of high phenolic content. Anthocyanins are one group of these polyphenols, and it both contributes to astringency and provide the reddish-purple color. Fermentation allows the enzymatic breakdown of proteins and carbohydrates inside the bean, creating flavor development. This is aided by microbial fermentation, which create the perfect environment through the fermentation of the cacao pulp surrounding the beans. This processing step enables the extraction of flavor from cacao and contributes to the final acidity of the final product. Yeasts (and also bacteria) ferment the juicy pulp among the cacao beans by different methods, generally following a an anaerobic phase and an aerobic phase. During the anaerobic phase, the sugars of the pulp (sucrose, glucose, fructose) are consumed by yeasts using anaerobic respiration to yield carbon dioxide, ethanol, and low amounts of energy [18,19]. The aerobic stage is dominated by lactic and acetic-acid-producing bacteria [20].

### 2.3. Not Only Food: Biofuels and Other Chemicals

The fermentation processes of substrates such as xylose are also of high interest on an industrial level. In addition to expanding the range of substrates that can be used for this purpose, they allow the environmental cost of efficient production of biofuels and other advanced chemicals to be reduced. Some interesting approaches have been made in biorefinery to reprogram yeast for use in these bioprocesses [21,22,23].

## 3. Special Issue on “Yeast Fermentation”

This issue in *Microorganisms* aims to contribute to the update of knowledge regarding yeasts, regarding both basic and also applied aspects. Among the great contributions to this issue we have a manuscript devoted to the brewing industry and the recent isolation of the yeast *Saccharomyces eubayanus* [24]. The use of headspace solid-phase microextraction followed by gas chromatography-mass spectrometry (HS-SPME-GC-MS) has contributed to the production of volatile compounds in wild strains and to compare them to a commercial yeast. All these findings highlight the potentiality of this yeast to produce new varieties of beers. Haile et al. [17] have explored the possibility to identify and select pectinolytic yeasts that have potential use as a starter culture for coffee fermentation. Almost 30 isolates, eight of them with the ability to produce pectinase enzymes were identified and confirmed by using molecular biology techniques. A helpful bioinformatics tool (MEGA 6) was also used to generate phylogenetic trees able to determine the evolutionary relationship of yeasts obtained from their experiments. Biofuel production by recombinant *Saccharomyces cerevisiae* strains with essential genes and metabolic networks for xylose metabolism has been also reported [23]. The authors have shown that the deletion of cAMP phosphodiesterase genes PDE1 and PDE2 can increase xylose utilization. Moreover, the door is opened to provide new targets for engineering other xylose-fermenting strains. The utilization of xylose, the second most abundant sugar component in the hydrolysates of lignocellulosic materials, is a relevant issue. Understanding the relationship between xylose and the metabolic regulatory systems in yeasts is a crucial aspects where hexokinase 2 (Hxk2p) is involved [25]. All of these processes can be damaged if contaminated. Because most fermentation substrates are not sterile, contamination is always a factor to consider. With a very interesting approach, a genetically modified strain of *Komagataella phaffii* yeast was used for the use of glycerol as a base substance in lactate production. Polyactide, a bioplastic widely used in the pharmaceutical, automotive, packaging and food industries was produced. The disruption of the gene encoding arabitol dehydrogenase (ArDH) was achieved, which improves the production of lactic acid by *K. phaffii* as a biocatalyst [26]. Seo et al. [27] have developed and proposed alternative solutions to control contamination. This review includes information on industrial uses of yeast fermentation, microbial contamination and its effects on yeast fermentations. Finally, they describe strategies for controlling microbial contamination.
